# Targeting the EWS-ETS transcriptional program by BET bromodomain inhibition in Ewing sarcoma

**DOI:** 10.18632/oncotarget.6385

**Published:** 2015-11-25

**Authors:** Tim Hensel, Chiara Giorgi, Oxana Schmidt, Julia Calzada-Wack, Frauke Neff, Thorsten Buch, Felix K. Niggli, Beat W. Schäfer, Stefan Burdach, Günther H.S. Richter

**Affiliations:** ^1^ Laboratory for Functional Genomics and Transplantation Biology, Children's Cancer Research Centre and Department of Pediatrics, Klinikum rechts der Isar, Technische Universität München, Munich, Germany; ^2^ Comprehensive Cancer Center Munich (CCCM), Munich, Germany; ^3^ Department of Oncology and Children's Research Center, University Children's Hospital, Zurich, Switzerland; ^4^ Institute of Pathology, Helmholtz Zentrum München - German Research Center for Environmental Health (GmbH), Neuherberg, Germany; ^5^ Institute for Medical Microbiology, Immunology and Hygiene, Technische Universität München, Munich, Germany; ^6^ Institute of Laboratory Animal Science, University of Zurich, Zurich, Switzerland

**Keywords:** BET bromodomains, Ewing sarcoma, tumor growth, JQ1, PI3K pathway

## Abstract

Ewing sarcomas (ES) are highly malignant bone or soft tissue tumors. Genetically, ES are defined by balanced chromosomal *EWS/ETS* translocations, which give rise to chimeric proteins (EWS-ETS) that generate an oncogenic transcriptional program associated with altered epigenetic marks throughout the genome. By use of an inhibitor (JQ1) blocking BET bromodomain binding proteins (BRDs) we strikingly observed a strong down-regulation of the predominant EWS-ETS protein EWS-FLI1 in a dose dependent manner. This was further enhanced by co-treatment with an inhibitor of the PI3K pathway. Microarray analysis further revealed JQ1 treatment to block a typical ES associated expression program. The effect on this expression program was mimicked by RNA interference with BRD3 or BRD4 expression, indicating that the EWS-FLI1 mediated expression profile is at least in part mediated via such epigenetic readers. Consequently, contact dependent and independent proliferation of different ES lines was strongly inhibited. Mechanistically, treatment of ES resulted in a partial arrest of the cell cycle as well as induction of apoptosis. Tumor development was suppressed dose dependently in a xeno-transplant model in immune deficient mice, overall indicating that ES may be susceptible to treatment with epigenetic inhibitors blocking BET bromodomain activity and the associated pathognomonic EWS-ETS transcriptional program.

## INTRODUCTION

Ewing sarcoma (ES) is a highly malignant bone and soft tissue neoplasia of still enigmatic histogenesis with a prominent *stemness* phenotype [1, 2]. Histogenesis may be endothelial, neuroectodermal [3-5] or osteo-chondrogenic [6, 7]. ES are characterized by early metastasis into lung and bone tissues. Metastasis is commonly haematogenous and related to *stemness* [1, 4, 8]. Even though prognosis for ES patients has markedly improved with the development of multimodal therapeutic approaches, the survival rate of patients with advanced, multifocal disease is still associated with fatal outcome [9-11]. Especially multifocal bone or bone marrow disease and the development of metastases in bones are catastrophic events in the clinical course of ES patients [9, 12]. Genetically, ES is defined by specific balanced chromosomal *EWS/ETS* translocations which give rise to oncogenic chimeric proteins, the most common being EWS-FLI1 as a consequence of the t(11;22)(q24;q12) translocation [13-15]. Other contributing somatic mutations involved in disease development have only been observed at low frequency [16-19].

Thus, cancer in children is not solely a genetic disease and can neither be understood nor cured presumably without epigenetics. We previously identified the histone methyl-transferase Enhancer of Zeste, Drosophila, Homolog 2 (EZH2), the enzymatic subunit of the polycomb PRC2 complex, to be over-expressed and regulated as a downstream event via EWS-FLI1 in ES. RNA interference of EZH2 suppressed tumor development and metastasis *in vivo* and microarray analysis of EZH2 knock down revealed an EZH2-maintained, undifferentiated, reversible phenotype in ES [1]. EZH2 suppression resulted in a generalized loss of H3K27me3 as well as increase in H3 acetylation. ChIP-Chip assays for H3K27me3 verified such genes that had specifically lost H3K27me3 upon EZH2 silencing [8], suggesting that malignant *stemness* features are preserved via epigenetic mechanisms. Recent results further indicate that EWS-ETS proteins not only deregulate components of the epigenetic machinery in ES [1], but in addition create specific epigenetic marks [20, 21] that might be addressed by epigenetic therapy.

BET proteins (BRD2, BRD3, BRD4, and the testis-specific BRDT) are bromodomain (BRD) containing proteins that belong to the bromo and extraterminal (BET) subset of BRD proteins. They are nuclear proteins that carry 2 bromodomains and an additional ET domain, and are implicated in chromatin interactions [22]. They seem to associate with transcription complexes and with acetylated chromatin [23]. Specific inhibitors of BET proteins such as I-BET151 or JQ1 resulted in displacement of BRDs from chromatin and inhibition of transcription at key genes such as BCL2, MYC, and CDK6 [23]. Initially it was shown that JQ1 could block the growth of a rare, aggressive form of human squamous carcinoma with BRD4-NUT translocation [24] as well as of MYC transformed multiple myeloma [25]. Effectivity of JQ1 and inhibition of C-MYC or N-MYC was also demonstrated for AML [26] or neuroblastoma [27], respectively.

In addition to BET inhibitors, also enhanced activity of the phosphoinositide 3-kinase (PI3K) pathway has been linked to MYC turnover [28] and thereby might potentially enhance the activity of BET inhibitors. Indeed, PI3K inhibition has been suggested as therapeutic option in ES before [29] and recent evidence suggests that the pathway can modulate expression of the EWS-FLI1 fusion protein itself [30]. By use of the BET bromodomain inhibitor JQ1 we significantly blocked proliferation and *in vivo* tumor growth of different ES lines and strikingly observed a strong down-regulation of the pathognomonic EWS-FLI1 protein. Subsequent analysis revealed that JQ1 treatment blocked an ES specific expression program and enhanced apoptosis of treated cell lines.

## RESULTS

### JQ1 blocks EWS-FLI1 expression in ES

**Figure 1:**
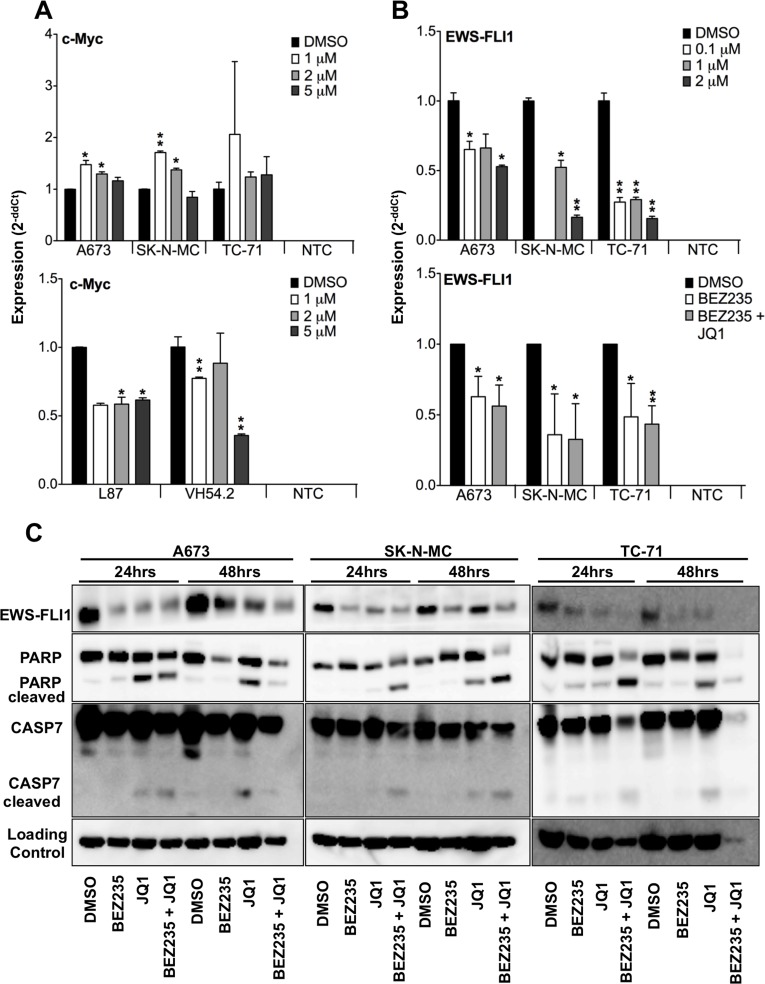
Blockade of BET bromodomain proteins blocks EWS-FLI1 but not MYC expression **A**. Top, MYC expression in ES cell lines A673, SK-N-MC and TC-71 and, bottom, in mesenchymal stem cells L87 and VH54.2 after 48hrs JQ1 treatment as measured by qRT-PCR. Data are mean ± SEM; t-test. NTC: non-template control. **B**. Top, different doses of JQ1 inhibit EWS-FLI1 expression in ES cell lines A673, SK-N-MC or TC-71, respectively. Bottom, relative expression of EWS-FLI1 measured by qRT-PCR in A673, SK-N-MC and TC-71 cells after 24hrs treatment with 500nM BEZ235 and 500nM BEZ235 in combination with 2μM JQ1 compared to DMSO control. Data are mean ± SEM; t-test. NTC: non-template control. **C**. Protein level measured by western blot of EWS-FLI1, PARP, CASP7 and loading control. Cells were treated for 24 and 48hrs with 500nM BEZ235, 2μM JQ1, 500nM BEZ235 in combination with 2μM JQ1 compared to DMSO control in A673, SK-N-MC and TC-71 cells. Shown is a representative experiment (n=3). *P-value < 0.05; **P-value < 0.005.

In a previous microarray analysis we identified the proto-oncogene MYC as being persistently up-regulated in ES (Supplementary Figure S1). To analyze the relevance of its expression, we employed the potent BET bromodomain inhibitor JQ1 and the PI3K inhibitor BEZ235 as possible pathways regulating MYC expression in ES cells and compared their impact to such on mesenchymal stem cells (MSCs). None of the analyzed ES cells showed any down-regulation of MYC expression after treatment with different concentrations of JQ1 (Figure 1A, Top) while in contrast MSCs showed an up to 65% down-regulation after 5μM JQ1 treatment in VH54.2 cells (Figure 1A, Bottom). Similarly, also BEZ235 treatment revealed no influence on MYC expression in ES (data not shown). Therefore, we became curious whether the characteristic oncofusion protein EWS-FLI1 is involved in MYC regulation and analyzed its expression upon treatment (Figure 1B and Supplementary Figure S2A). Surprisingly, expression of EWS-FLI1 was reduced after either just JQ1 treatment or combined JQ1 and BEZ235 treatment, which was also confirmed at protein level (Figure 1C). Furthermore, PARP1 and caspase 7 cleavage (Figure 1C) increased after 24 as well as 48 hours JQ1 or combined JQ1 and BEZ235 treatment, indicating induction of apoptosis of ES cells especially after JQ1 and combination treatment.

### JQ1 down-regulates an ES specific expression profile

**Figure 2:**
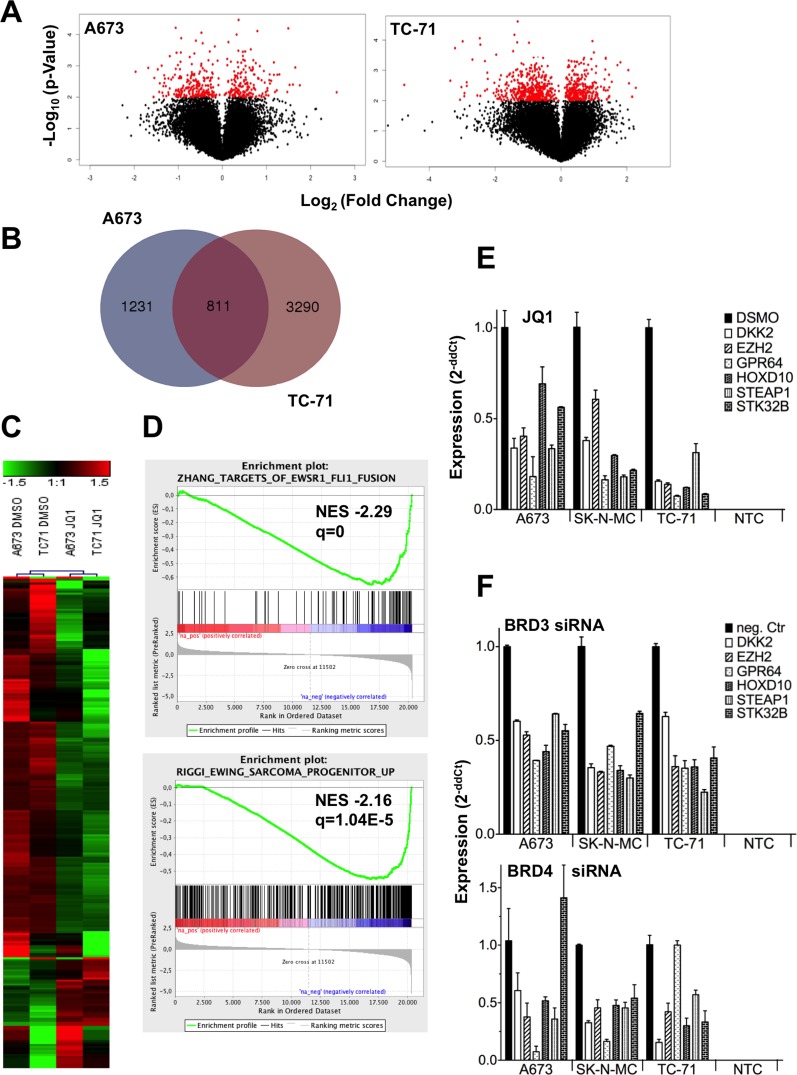
ES expression profile after JQ1 treatment or RNA interference of BRD genes blocks a typical ES associated expression program **A**. Volcano plot for DMSO against JQ1 treated ES lines, showing the adjusted significance *P*-value (−log_10_) plotted over fold change (log2). Red, genes with a significance *P* < 0.01. Microarray data with their normalized fluorescent signal intensities were used (RMA, see *Materials and Methods;* GSE72673). Cells were treated with DMSO or JQ1 for 48hrs, collected, and then analyzed. **B**. Shared genes differentially expressed after JQ1 treatment in 2 different ES lines. For a Venn diagram genes ± 1.5 fold differentially expressed were selected for the analysis (http://bioinformatics.psb.ugent.be/webtools/Venn/). **C**. Heat map of 244 genes, 1.8-fold differentially expressed in 2 different ES lines A673 and TC-71 are shown. Each column represents 1 individual array. **D**. GSEA enrichment plots of down-regulated genes after JQ1 treatment. GSEA: http://www.broadinstitute.org/gsea/index.jsp **E**. Verification of microarray data by qRT-PCR of selected genes. ES specific genes were significantly down-regulated after JQ1 treatment in different ES cell lines. **F**. RNA interference of BRD3 or BRD4, respectively with specific siRNAs affects the same ES specific genes as after JQ1 treatment. Results of qRT-PCRs are shown. Data are mean ± SEM; t-test. NTC: non-template control.

To clarify to which extent JQ1 influences gene expression in ES cells, we carried out microarray analyses on JQ1 treated A673 and TC-71 cells. Analysis of differentially expressed genes using volcano plots indicated 720 and 405 genes significantly up- and down-regulated upon treatment in TC-71 and A673, respectively (P-value < 0.01; Figure 2A). Comparison of expression data between both cell lines at a fold change ± 1.5 by Venn analysis (http://bioinformatics.psb.ugent.be/webtools/Venn/) revealed 811 shared, differentially expressed genes (Figure 2B). Further, a heat map of 244 differentially expressed genes in both cell lines (fold change > 1.8) is shown (Figure 2C). Most of the identified genes were down-regulated after JQ1 treatment (188 genes were down-regulated, 57 were up-regulated, GSE72673). Subsequent gene set enrichment analysis (GSEA) identified a down-regulation of gene sets typical for EWS-FLI1 fusion targets as identified by Zhang and colleagues [31] as well as those for Ewing sarcoma progenitors identified by Riggi *et al.* [32] (Figure 2D), indicating that JQ1 inhibits EWS-FLI1 expression and thereby similarly inhibits an ES typical expression profile (Supplementary Table S1). Such genes including *DKK2*, *EZH2*, *GPR64*, *PAPPA*, *STEAP1*, and *STK32B* were shown to be consistently up-regulated and demonstrated to be involved in ES pathogenesis [1, 6, 8, 33, 34]. They were verified to be down-regulated by JQ1 treatment using qRT-PCR, overall confirming results of the microarray analysis (Figure 2E).

### RNA interference of different BRDs by specific siRNAs mimics the JQ1 treatment effect in ES

*BRD2*, *BRD3*, *BRD4* but not *BRDT* genes are well expressed in ES (Supplementary Figure S1). While JQ1 has been reported to be most specific for BRD4 protein, binding to the remaining BRD proteins was also observed, although to a lesser extent [24]. Therefore BRD2, 3 or 4 were transiently down-regulated by specific siRNA in ES and their influence on an ES specific expression profile was analyzed. While BRD2 knock down did not result in any expression changes on such genes (Supplementary Figure S2B), BRD3 and BRD4 knock down uniformly resulted in a similar down-regulation of ES specific genes (Figure 2F) as observed after JQ1 treatment (Figure 2E), concluding that BRD3 as well as BRD4 might be the essential targets of JQ1 treatment hereby repressing the pathognomonic EWS-FLI1 driven expression profile.

### JQ1 treatment inhibits proliferation, cell cycle progression and promotes apoptosis

**Figure 3:**
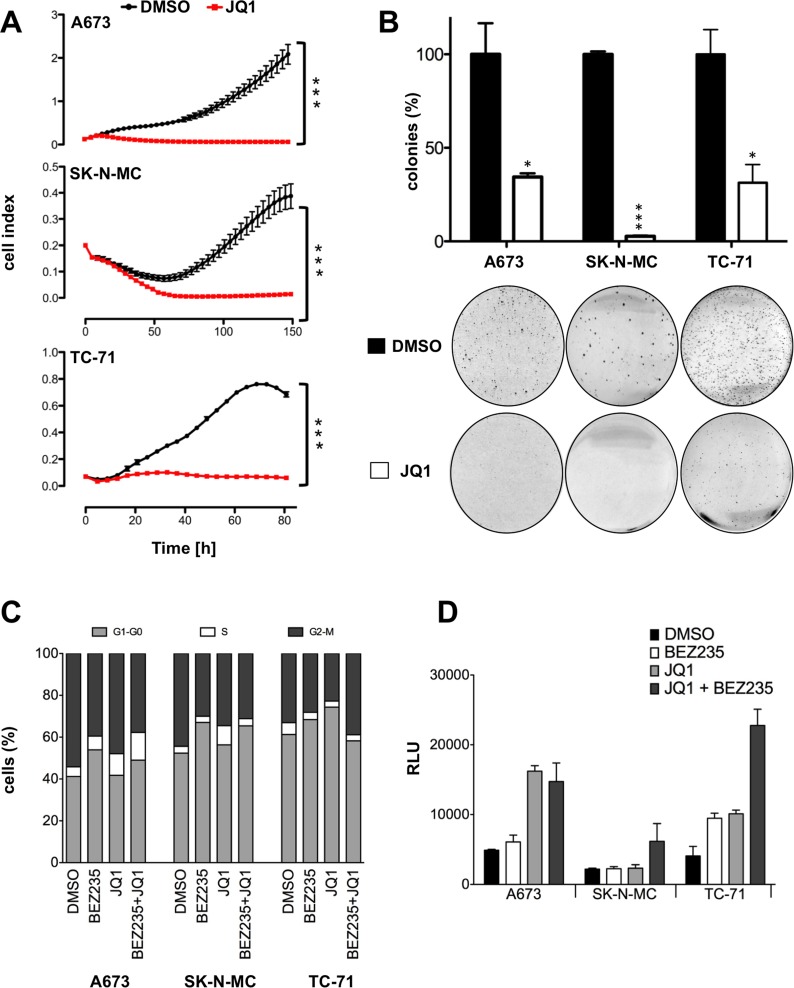
Treatment with JQ1 blocks proliferation, cell cycle progression and induces caspase dependent apoptosis **A**. Analysis of proliferation of JQ1 treated ES cell lines in comparison to vehicle with xCELLigence. Cellular impedance was measured every 4hrs (relative cell index). Data are mean ± SEM (hexaplicates/group); t-test. **B**. Analysis of anchorage-independent colony formation in methylcellulose of ES lines after JQ1 treatment. Top, data are mean ± SEM of 3 independent experiments (duplicates/group); t-test. Bottom, macrographs show a representative experiment with A673, SK-N-MC and TC-71. **C**. Cell cycle progression upon JQ1 and BEZ235 treatment. Cell cycle analysis after 24 and 48hrs treatment with 500nM BEZ235, 2μM JQ1, 500nM BEZ235 in combination with 2μM JQ1 compared to DMSO treated control in A673, SK-N-MC and TC-71 cells. Shown is a representative experiment (n=3). **D**. Caspase 3/7 activity measured after 24hrs treatment with 500nM BEZ235, 2μM JQ1, 500nM BEZ235 in combination with 2μM JQ1 in A673, SK-N-MC and TC-71. Bars represent mean values expressed as relative light unit (RLU) in percentage of DMSO treated control of 6 biological replicate analyzed in three technical replicates each (n=3; SE<0.01). ***P-value < 0.0005.

Based on these results, we asked whether the inhibition of the EWS-FLI1 specific expression profile may also affect the growth abilities of ES. Using the xCELLigence assay, we compared contact dependent growth of different ES lines A673, SK-N-MC and TC-71 either treated with 2μM JQ1 or just DMSO (Figure 3A). Neither of the analyzed cells showed a significant increase of cell numbers after JQ1 application. Similarly, contact independent growth of JQ1 treated A673, SK-N-MC and TC-71 revealed a strong reduction of colony formation (Figure 3B) in methylcellulose assay. We subsequently asked whether this reduction of proliferative capacity might be due to changes in cell cycle progression. Flow cytometry analysis for all 3 cell lines depicted a reduction of G2-M phase while an increase in G1 phase was observed after JQ1 treatment in SK-N-MC and TC-71 cells (Figure 3C) as well as an extension of S phase in A673 and SK-N-MC cells. BEZ235 treatment more consistently increased the G1 phase of all 3 cell lines and had no additional effect on the cell cycle when combined with JQ1 (Figure 3C). In addition, the caspase 3 glow assay revealed an increase of apoptosis after JQ1 treatment in A673 and TC-71 that was further increased by combined treatment with BEZ235 in SK-N-MC cells (Figure 3D). These results together with the observed PARP1 and caspase 7 cleavage (Figure 1C) indicate a strong effect of JQ1 on apoptosis induction in ES.

### JQ1 reduces tumor growth *in vivo* in a dose dependent manner

**Figure 4:**
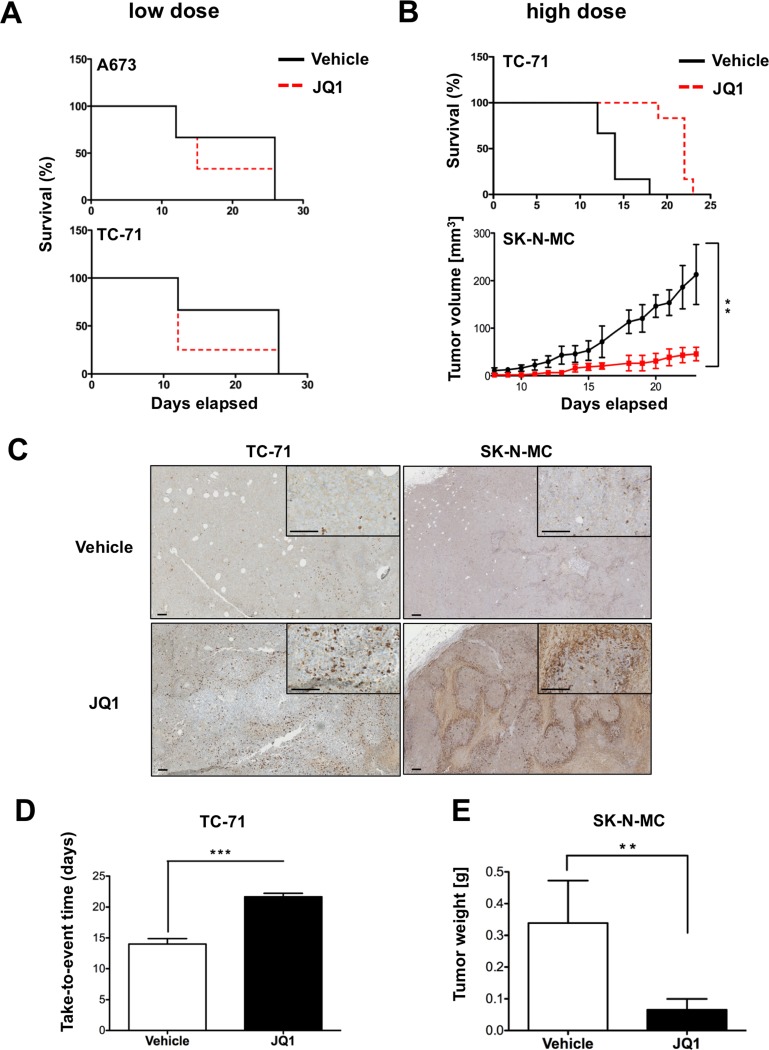
Treatment with JQ1 inhibits ES tumor growth *in vivo* Evaluation of the therapeutic potential of JQ1 application. Immune deficient Rag2^−/−^γc^−/−^ mice were injected s.c. with 2×10^6^ ES cells. 5-7 days later these mice received different doses of JQ1 or vehicle i.p., respectively. Delay or inhibition of tumor growth was evaluated. **A**. Mice were either injected with A673 or SK-N-MC cells and 7 days later received 50 mg/kg JQ1 or vehicle every other day. Mice with an average tumor size >10 mm in diameter were considered as positive and killed. Kaplan–Meier plots of individual experiments with 5 mice per group are shown. **B**. Mice were injected with tumor cells s.c. and 5 days later received twice daily doses for 14 to 23 days for TC-71 and SK-N-MC, respectively. Top, survival of TC-71 inoculated mice. Bottom, tumor growth after inoculation with SK-N-MC cells. (6 mice/group). **C**. To analyze intratumoral changes after high dose JQ1 application tumors were collected upon tumor burden (TC-71) or after 23 days (SK-N-MC). The pictures show clear increased expression of cleaved caspase 3 in tumors treated with JQ1. Bar indicates 0.1 mm. **D**. Variation of tumor growth characteristics analyzed as a function of time until tumors reached >1 cm^3^ size for TC-71 inoculated mice. **E**. Determined tumor weight of SK-N-MC inoculated mice at the end of the experiment. **P-value < 0.005, ***P-value < 0.0005.

As already demonstrated by others [24-27], JQ1 may also have a therapeutic effect *in vivo*. Therefore we also evaluated the therapeutic suitability of ES cells to JQ1 treatment in a xenograft mouse model of Rag2^−/−^γc^−/−^ mice by implanting tumor cells s.c. into mice. Starting with 50 mg/kg body weight every other day by intraperitoneal injection of JQ1 or vehicle we observed no growth rate reduction on A673 or TC-71 cells, respectively (Figure 4A). Assuming that this concentration of JQ1 might be too low for a pharmaceutically effective supply over two days we also tested more frequent doses on TC-71 cells. Administration of 50 mg/kg twice daily for a period of 14 days resulted in an elongated survival of treated mice (Figure 4B, Top and 4D). To confirm these results we chose SK-N-MC due to its strict dependency on EWS-FLI1 expression. Mice were treated twice daily with 50 mg/kg for 23 days revealing a significant growth reduction (Figure 4B, bottom) and decreased tumor weight (Figure 4E). Further, tumors prepared at the end of the experiment and analyzed immunohistochemically for caspase 3 expression revealed an increased apoptosis rate in tumors treated with JQ1 (Figure 4C). However, further increase to 75 mg/kg twice daily was too toxic and resulted in severe weight loss and death of some mice (data not shown). Overall, both experiments demonstrated a significant inhibition of ES growth *in vivo* at higher JQ1 dosage and the potential therapeutic value of BET bromodomain inhibitors for the treatment of ES.

## DISCUSSION

In Ewing sarcoma (ES) the deregulation of components of the epigenetic machinery was previously demonstrated as an important step for tumor formation [1, 8]. Furthermore, it was recently shown that EWS-FLI1 employs divergent chromatin remodeling mechanisms to activate or repress transcription [20, 21]. Herein altered epigenetic marks were identified that generated specific acetyl-lysine moieties on histones and may be targeted by epigenetic reader proteins such as BET proteins (BRD2, BRD3, BRD4). Interestingly, all three BET proteins are well expressed in ES and may be targeted by specific inhibitors of BET proteins such as I-BET151 [23] or JQ1 [24]. Such treatment resulted in dislodgment of BRDs from chromatin in other tumors and inhibition of transcription at key genes involved in apoptosis, cell cycle regulation and oncogenesis [26, 27], [35] [36]. It was demonstrated that JQ1 could block tumor growth and a recurring feature of the consequences of JQ1 / I-BET151 treatment was inhibition of MYC, N-MYC or FOSL [37] expression, respectively in these tumors.

MYC over-expression is well known in ES. Its level of expression seems to be directly regulated via EWS-FLI1 [38]. By use of JQ1 in different ES cell lines we significantly blocked their proliferation and strikingly observed a strong down-regulation of the pathognomonic EWS-FLI1 protein. We saw no inhibition of MYC expression in ES lines whereas its inhibition was readily observed in MSC.

JQ1 treatment suppressed a number of genes typical for ES specific expression profiles [5, 8, 32]. For example GPR64, a new excellent marker of ES [33], was down-regulated after JQ1 treatment. Similarly, the expression of DKK2, a key player of ES invasiveness and osteolytic tumor growth [6], was greatly reduced by JQ1. Further, other genes consistently up-regulated and/or shown to be involved in ES pathogenesis such as *EZH2*, *PAPPA*, *STEAP1*, and *STK32B* [1, 8, 34] were uniformly inhibited by JQ1.

Consistently fewer genes were found to be up-regulated after JQ1 treatment and included genes involved mainly in pathways for cell maturation, differentiation, etc. (GSE72673), confirming that EWS-FLI1 itself is abrogating differentiation programs and is the driver of the immature phenotype of this disease [1, 29].

Interestingly, the JQ1 treatment effect on this expression program of ES lines was mimicked by specific siRNA-mediated knock down of BRD3 or BRD4 but not BRD2 expression, demonstrating that not only BRD4 is an important epigenetic reader protein in ES. Displacement of BRD3/4 by specific inhibitors has already been shown in MLL-fusion positive leukemia [23]. While BRD3 seems to preferentially associate with hyper-acetylated chromatin along the entire lengths of transcribed genes [39], BRD4-binding has also been observed in enhancer regions [40]. To what areas of chromatin BET proteins bind in ES has to be further investigated.

JQ1 treatment not only suppressed an ES specific expression profile but also blocked contact dependent and independent proliferation of different ES lines. This seems to be due to a partial G1 arrest and S phase elongation of the cell cycle as demonstrated previously [24]. In addition, induction of apoptosis as demonstrated by PARP1-, CASP7-cleavage and increased CASP3 activity seems to significantly contribute to the reduction of the proliferative ability of ES lines. Single or combination treatment with the PI3K/mTOR inhibitor BEZ235 [41] did increase apoptosis of ES cell lines although single treatment with BEZ235 was less effective than JQ1 application.

However, a number of substances initially also demonstrated efficacy in preclinical models such as single treatment with ARA-C [42] or anti-IGFR [43] but in phase I clinical trials delivered transient [29] or disappointing results [44], emphasizing the need to combine drugs that based on the biology of this tumor may result in synergistic growth inhibitory effects.

For example, treatment with BEZ235 clearly inhibited EWS-FLI1 expression and in combination with JQ1 further increased apoptosis induction indicating that combination treatment of JQ1 with PI3K/mTOR inhibition should be a promising strategy for future therapy of ES.

Also, combination treatment of JQ1 with substances like YK-4-279 that directly binds to EWS-FLI1 and inhibits its oncogenic activity [45, 46] via blockade of specific protein interaction with factors important for mRNA splicing [47] and transcription [45] may result in synergistic effects on tumor growth and needs to be explored in preclinical models of ES.

Similarly, combination treatment with EZH2 inhibitors such as GSK126 [48, 49] may further increase therapeutic efficacy and due to potential synergistic effects decrease JQ1 dose levels required for successful treatment of ES.

Here, we at first demonstrated that tumor development was dose dependently suppressed by intra-peritoneal JQ1 application in a xeno-transplant model of ES–bearing immune deficient Rag2^−/−^γc^−/−^ mice. Therapeutic efficacious doses, although high, were within the range published previously [24, 27, 35]. Overall, our results demonstrate that MYC or EWS-FLI1 mediated pathognomonic expression programs may be similarly targeted by BET bromodomain inhibition, casting BET protein inhibition appropriate as a potential platform for future combination therapy of this disease.

## MATERIALS AND METHODS

### Cell lines

ES lines (SK-N-MC and TC-71), neuroblastoma lines (CHP126, MHH-NB11, SHSY5Y and SIMA) were obtained from the German Collection of Microorganisms and Cell Cultures (DSMZ, Braunschweig, Germany). A673 was purchased from ATCC (LGC Standards, Teddington, UK). Mesenchymal stem cell lines L87 an V54.2 were described previously [33]. Cells were maintained in a humidified incubator at 37°C in 5-8 % CO_2_ atmosphere in RPMI 1640 (Life Technologies, Carlsbad, CA, USA) containing 10 % heat-inactivated fetal bovine serum (Biochrom, Berlin, Germany) and 100 μg/ml gentamicin (Life Technologies). Cell lines were checked routinely for purity (e.g. EWS-FLI1 translocation product, surface antigen or HLA-phenotype) and Mycoplasma contamination.

### RNA interference (RNAi)

For transient RNA interference cells were transfected with small interfering RNA (siRNA) as described previously [1]. To test transfection efficiency and gene silencing RNA was extracted and gene expression assessed by quantitative Real Time-PCR. siRNA sequences are provided in the Supplementary Information.

### Quantitative real time-PCR (qRT-PCR)

Total RNA was isolated and reverse transcribed using the High Capacity cDNA Reverse Transcription Kit (Thermo Fisher Scientific AG) according to the manufacturer's instructions. Differential gene expression was then analyzed by qRT-PCR using TaqMan Universal PCR Master Mix and ﬂuorescence detection with Step One Plus Real-Time PCR or ABI 7900 instrument (Thermo Fisher Scientific AG) as described previously [1, 33]. Gene expression was normalized to glyceraldehyde-3-phosphate dehydrogenase (GAPDH). All experiments were performed at least in duplicate for each cell line. A list of used assays is provided in the Supplementary Data. NTC: non template control.

### Proliferation assay

Cell proliferation was measured with an impedance-based instrument system (xCELLigence, Roche/ACEA Biosciences, Basel, Switzerland) enabling label-free real time cell analysis. Briefly, 4 - 10 × 10^3^ cells were seeded into 96-wells with 200 μl media containing 10 % FBS and allowed to grow up to 150 hours. Cellular impedance was measured periodically every 4 hours and 2μM JQ1 or DMSO was added.

### Colony forming assay

Cells were seeded in duplicate into a 35 mm plate at a density of 5 × 10^3^ cells per 1.5 ml methylcellulose-based media (R&D Systems, Minneapolis, MN, USA) according to the manufacturer's instructions and cultured for 10-14 days at 37°C / 5 % CO_2_ in a humidified atmosphere. 2μM JQ1 or DMSO was added

### Immunoblotting

5 × 10^5^ A673, SK-N-MC or TC-71 cells were treated with 500nM BEZ235, 2μM JQ1, 1μM JQ1, 500nM BEZ235 in combination with 2μM JQ1 or DMSO as controls, washed twice with PBS and harvested in lysis buffer containing 50mM NaH_2_PO_4_ (pH 7.5), 150mM NaCl, 1% Triton X-100, 1mM Na3OV4, 5mM Na-pyrophosphate, 40nM NaF, 1mM EGTA supplemented with protease inhibitor cocktail (Complete + 1mM EDTA, Roche Diagnostics AG). Protein concentration was determined by BCA (Thermo Fisher Scientific AG). 10–30μg of protein extract was resolved on 4-12% SDS-PAGE and transferred onto nitrocellulose membrane (Thermo Fisher Scientific AG). Primary antibodies were used as follows: anti-FLI1 monoclonal antibody (MyBioSource LLC, San Diego, USA), anti-PARP rabbit polyclonal antibody (Cell Signaling Technology, Danvers, USA), anti-Caspase7 antibody (Cell Signaling) and loading control (anti-β-tubulin (Sigma-Aldrich, St. Louis, USA) or GAPDH (Cell Signaling)). After incubation with the appropriate secondary peroxidase-conjugated antibodies, detection was performed with the ECL chemiluminescence reagent (Amersham Biosciences, Little Chalfont, UK).

### FACS analysis

Treated cells were washed with PBS, collected, fixed with 70% Ethanol for 2hrs on ice and stained with PI solution- for 1hr at room temperature- 20 μg/ml PI (Sigma-Aldrich), PBS, 0.1% TritonX, 200 μg/ml RNAse A for measurement with a FACS Canto. Data were analyzed using Flow Jo program (Flow Jo LLC., Ashland, OR, USA).

### Casp3/7 assay

4 × 10^3^ A673, SK-N-MC or TC-71 cells, were plated in a 384-well plate previously coated with 0.2% gelatin. After 24hrs, cells were treated with 500nM BEZ235, 2μM JQ1, 500nM BEZ235 in combination with 2μM JQ1 or DMSO as controls. 24hrs after treatment Caspase 3/7 reagent (Promega AG) was added in each well and luminescence was measured.

### Microarray analysis

Biotinylated target cRNA was prepared as previously described [1]. A detailed protocol is available at www.affymetrix.com. Samples were hybridized to Affymetrix Human Gene 1.0 ST microarrays and analyzed by Affymetrix software expression console, version 1.1. For the data analysis, robust multichip average (RMA) normalization was performed, including background correlation, quantile normalization, and median polish summary method. Probes of the normal body map (NBA) included tissues of normal PBMC, bone marrow, spleen, thymus, stomach (2), small intestine, colon w/ mucosa, heart, liver, lung, skeletal muscle, brain (whole), brain cerebellum, spinal cord, trachea, salivary gland, prostate, testis, uterus, fetal brain, and fetal liver. Array data were submitted at GEO (GSE45544).

### Animal model

Immune deficient Rag2^−/−^γc^−/−^ mice on a BALB/c background were obtained from the Central Institute for Experimental Animals (Kawasaki, Japan) and maintained in our animal facility under pathogen-free conditions in accordance with the institutional guidelines and approval by local authorities (Regierung von Oberbayern). Experiments were performed in 6-20 week old mice.

### *In vivo* experiments

To examine *in vivo* tumorigenicity, 2-3 × 10^6^ ES cells were injected subcutaneously into the inguinal region of immune deficient Rag2^−/−^γc^−/−^ mice. JQ1 was handled and dissolved as recommended by the Bradner lab and administered at 50 mg/kg body weight intra peritoneal either twice daily or every other day. Mice were monitored daily and tumor xenografts were measured with digital calipers, and tumor volume was calculated as (L x W^2^) / 2, where L is length and W is width. Experimental endpoints were determined by completion of treatment or attainment of tumor burden exceeding 1 cm^3^. Upon reaching endpoints, mice were humanely euthanized and tumors excised and characterized.

### Histology

Histological analysis of tumor specimens was performed in a minimum of 5 mice per group. Tissues organs were fixed in phosphate buffered 4% formaldehyde and paraffin embedded. 3-5μm thick sections from all tissues were stained with hematoxylin and eosin (H&E). Apoptosis was evaluated by immunohistochemistry (IHC) using cleaved Caspase 3 (Cell Signaling) as primary antibody. The IHC was performed using the streptavidin–peroxidase method with an automated immunostainer (DiscoveryXT; Roche, Penzberg, Germany), All sections were reviewed and interpreted by two pathologists.

### Statistical analyses

Data are mean ± SEM as indicated. Differences were analyzed by unpaired two-tailed student's t-test as indicated using Excel (Microsoft, Redmond, WA, USA) or Prism 5 (GraphPad Software, San Diego, CA, USA); P-values < 0.05 were considered statistically significant (*p < 0.05; **p < 0.005; ***p < 0.0005). Volcano plots were drawn using R, a free software environment available at http://www.r-project.org/.

## SUPPLEMENTARY MATERIAL FIGURES AND TABLE


