# Supplementary material for: Targeting the EWS-ETS transcriptional program by BET bromodomain inhibition in Ewing sarcoma
Source: Oncotarget. 2015 Nov 25;7(2):1451–63. doi: 10.18632/oncotarget.6385 (PMC4811472; doi:10.18632/oncotarget.6385)
Supplement: Supplementary file 1 [file oncotarget-07-1451-s001.pdf]

Targeting the EWS-ETS transcriptional program by BET bromodomain inhibition in Ewing sarcoma

Supplementary Material

Methods

Small interfering RNAs used:

All siRNAs were purchased from Qiagen. Sequences are as follows: siBRD2_8 5'-GUAGCAGUGUCACGCCUUATT-3' (sense) and 5'-UAAGGCGUGACACUGCUACTT-3' (antisense); siBRD3_8 5'-GCCGCCUGUCGUCAAGAAATT-3' (sense) and 5'-UUUCUUGACGACAGGCGGCGT-3' (antisense); siBRD4_9 5'-GGACUAGAAACUUCCCAAATT-3' (sense) and 5'-UUUGGGAAGUUUCUAGUCCAT-3' (antisense) and control non silencing siRNA 5'-UUCUCCGAACGUGUCACGU-3' (sense) and 5'-ACGUGACACGUUCGGAGAA-3' (antisense).

Primers and assays used for RT-PCR

For EWS-FLI1 detection, the following primers 5'-TAGTTACCCACCCCAAACTGGAT-3' (sense) 5'-GGGCCGTTGCTCTGTATTCTTAC-3' (antisense) and probe 5'-FAM CAGCTACGGGCAGCAGAACCCTTCTT-TAMRA-3' were designed. Inventoried TaqMan Gene Expression Assays (Thermo Fisher Scientific AG) were used for the genes *DKK2* (Hs00205294_m1), *GAPDH* (Hs99999905_m1), EZH2 (Hs01016789_m1), BRD2 (Hs01121986_g1), BRD3 (Hs00201284_m1), BRD4 (Hs04188087_m1), STEAP1 (Hs00185180_m1), GPR64 (Hs00971379_m1), HOXD10 (Hs00157974_m1), STK32B (Hs00179683_m1), c-Myc (Hs00153408_m1) .

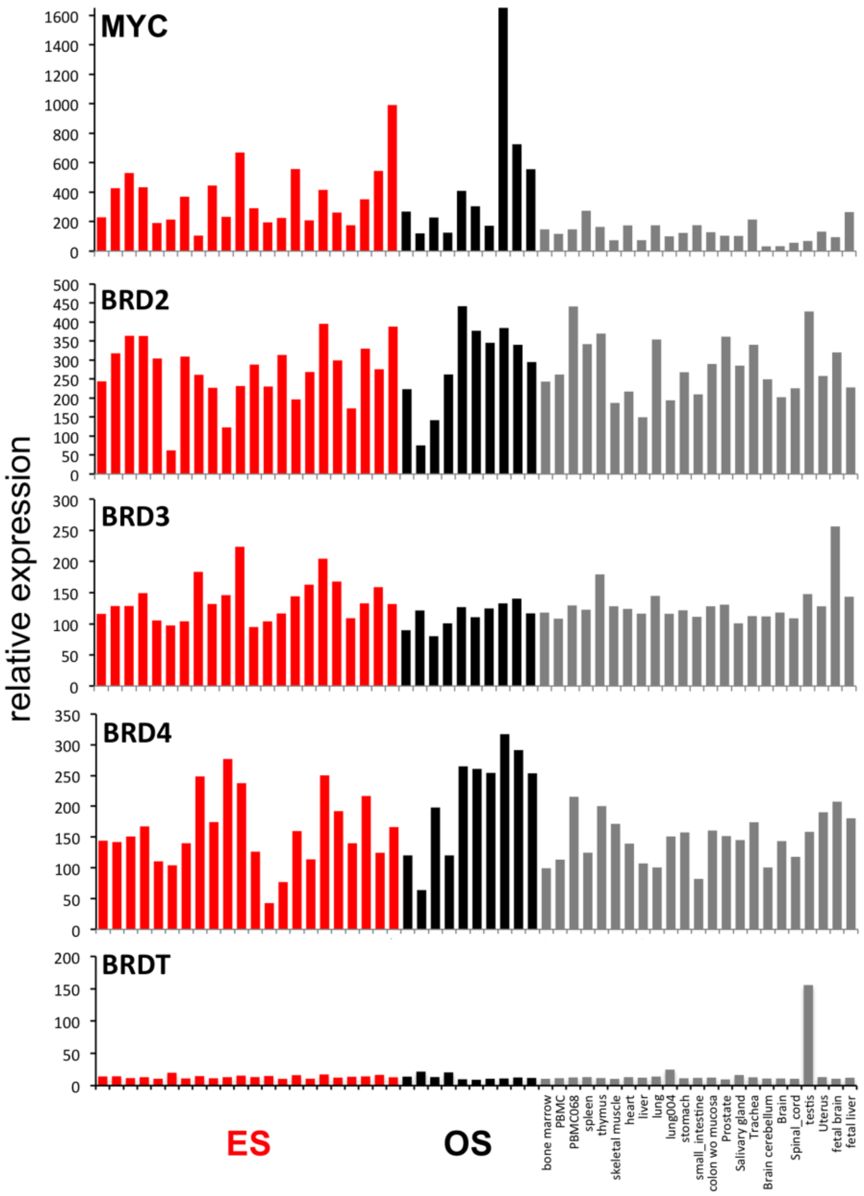

Supplementary Figure S1. Microarray analysis of primary Ewing and Osteosarcoma. Tumor RNA was hybridized onto Human Gene 1.0 ST microarrays (Affymetrix; Santa Clara, CA), analyzed by Affymetrix software expression console, version 1.1. and compared to normal tissue (GSE45544, GSE73166). Signal intensities in Ewing and osteosarcoma in comparison to normal tissue for MYC and BET genes (BRD2, BRD3, BRD4, BRDT) are shown.

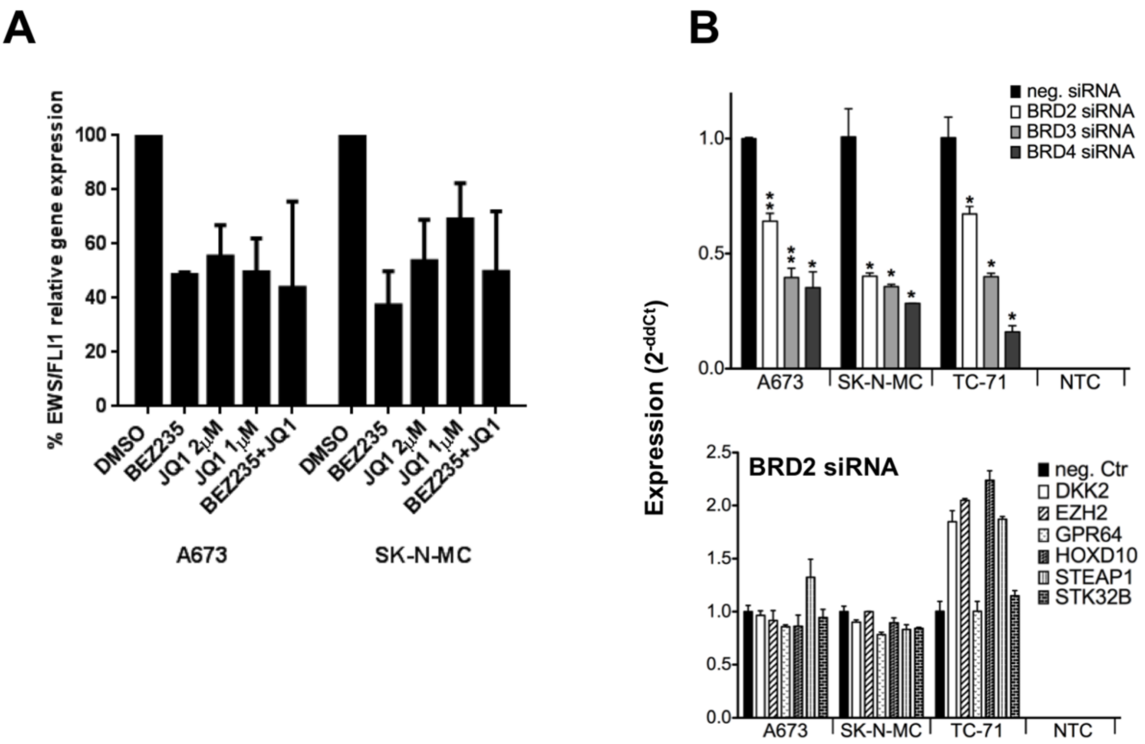

Supplementary Figure S2. JQ1 and BEZ235 treatment reduces EWS-FLI1 expression *in vitro*. A. Relative expression of EWS-FLI1 measured by qRT-PCR after 24hrs treatment with 500nM BEZ235, 2µM JQ1, 1µM JQ1 and 500nM BEZ235 in combination with 2µM JQ1 compared to DMSO control in SK-N-MC and A673 cells. Shown are representative experiments (n=3). **B**. Relative Expression of BRD2, 3 or 4 after 24 - 66hrs of RNA interference determined by qRT-PCR and analysis of JQ1 regulated genes after BRD2 knock down.

Supplementary Table S1*

Gene Symbol	Gene Description	Q-JQ1
GPR64	G protein-coupled receptor 64	0.243
JMJD1C	jumonji domain containing 1C	0.249
SMARCAD1	SWI/SNF-related, matrix-associated actin-dependent regulator of chromatin, subfamily a, containing DEAD/H box 1	0.275
STAG2	stromal antigen 2	0.283
LYN	v-yes-1 Yamaguchi sarcoma viral related oncogene homolog	0.288
PAPPA	pregnancy-associated plasma protein A, pappalysin 1	0.313
PCDHB5	protocadherin beta 5	0.325
IDH1	isocitrate dehydrogenase 1 (NADP+), soluble	0.334
STEAP1	six transmembrane epithelial antigen of the prostate 1	0.367
HDAC9	histone deacetylase 9	0.371
RGS4	regulator of G-protein signaling 4	0.380
CCNA1	cyclin A1	0.382
STEAP2	six transmembrane epithelial antigen of the prostate 2	0.388
HOXB2	homeobox B2	0.393
TET2	tet oncogene family member 2	0.405
IGF2BP1	insulin-like growth factor 2 mRNA binding protein 1	0.425
HIST1H2AB	histone cluster 1, H2ab	0.427
LIPI	lipase, member I	0.436
HIST1H1T	histone cluster 1, H1t	0.442
HOXD10	homeobox D10	0.445
HIST1H2BB	histone cluster 1, H2bb	0.448
CCNB2	cyclin B2	0.451
JARID2	jumonji, AT rich interactive domain 2	0.458
HDAC8	histone deacetylase 8	0.470
TET1	tet oncogene 1	0.473
TET2	tet oncogene family member 2	0.477
STK32B	serine/threonine kinase 32B	0.480
DKK2	dickkopf homolog 2 (Xenopus laevis)	0.480
SMARCAL1	SWI/SNF related, matrix associated, actin dependent regulator of chromatin, subfamily a-like 1	0.482
CCNA2	cyclin A2	0.483
JHDM1D	jumonji C domain containing histone demethylase 1 homolog D (S. cerevisiae)	0.492
EZH2	enhancer of zeste homolog 2 (Drosophila)	0.495
PAX7	paired box 7	0.499

*Selected genes down-regulated after JQ1 inhibition in TC-71 cells are shown.
